# Lack of mitochondrial complex I assembly factor NDUFAF2 results in a distinctive infantile-onset brainstem neurodegenerative disease with early lethality

**DOI:** 10.1186/s13023-024-03094-0

**Published:** 2024-02-28

**Authors:** Firas Abu Hanna, Yoav Zehavi, Eran Cohen-Barak, Morad Khayat, Nasim Warwar, Roni Shreter, Richard J. Rodenburg, Ronen Spiegel

**Affiliations:** 1 Department of Pediatrics B, Emek Medical Center, 1834111 Afula, Israel; 2 Rappaport School of Medicine, Technion, Haifa, Israel; 3 Department of Dermatology, Emek Medical Center, Afula, Israel; 4 Emek Medical Center, Genetic Institute, Afula, Israel; 5 Neuroradiology Unit, Hilel Yaffe Medical Center, Hadera, Israel; 6 Translational Metabolic Laboratory, Departments of Pediatrics and Genetics, Radboud UMC, Nijmegen, The Netherlands

**Keywords:** Mitochondrial disease, *NDUFAF2* gene, Leigh syndrome, Oxidative phosphorylation, Optic neuropathy

## Abstract

**Background:**

Congenital disorders of the mitochondrial respiratory chain are a heterogeneous group of inborn errors of metabolism. Among them, NADH:ubiquinone oxidoreductase (complex I, CI) deficiency is the most common. Biallelic pathogenic variants in *NDUFAF2*, encoding the nuclear assembly CI factor NDUFAF2, were initially reported to cause progressive encephalopathy beginning in infancy. Since the initial report in 2005, less than a dozen patients with *NDUFAF2*-related disease have been reported.

**Methods:**

Clinical, biochemical, and neuroradiological features of four new patients residing in Northern Israel were collected during 2016–2022 at Emek Medical Center. Enzymatic activities of the five respiratory-chain complexes were determined in isolated fibroblast mitochondria by spectrophotometric methods. Western blot analyses were conducted with anti-human NDUFAF2 antibody; antibody against the mitochondrial marker VDAC1 was used as a loading control. Genetic studies were performed by chromosome microarray analysis using Affymetrix CytoScan 750 K arrays.

**Results:**

All four patients presented with infantile-onset growth retardation, ophthalmological impairments with nystagmus, strabismus (starting between 5 and 9 months), and further progressed to life-threatening episodes of apnea usually triggered by trivial febrile illnesses (between 10 and 18 months) with gradual loss of acquired developmental milestones (3 of 4 patients). Serial magnetic-resonance imaging studies in two of the four patients showed a progressive pattern of abnormal T2-weighted hyperintense signals involving primarily the brainstem, the upper cervical cord, and later, the basal ganglia and thalami. Magnetic-resonance spectroscopy in one patient showed an increased lactate peak. Disease progression was marked by ventilatory dependency and early lethality. 3 of the 4 patients tested, harbored a homozygous 142-kb partial interstitial deletion that omits exons 2–4 of *NDUFAF2*. Mitochondrial CI activity was significantly decreased in the only patient tested. Western blot analysis disclosed the absence of NDUFAF2 protein compared to normal controls. In addition, we reviewed all 10 previously reported NDUFAF2-deficient cases to better characterize the disease.

**Conclusions:**

Biallelic loss-of-function mutations in *NDUFAF2* result in a distinctive phenotype in the spectrum of Leigh syndrome with clinical and neuroradiological features that are primarily attributed to progressive brainstem damage.

## Introduction

Congenital disorders of the mitochondrial respiratory chain are a heterogeneous group of inborn errors of metabolism with an estimated incidence of 1:5000–1:8000 live births. Among these, NADH:ubiquinone oxidoreductase (complex I, CI) deficiency is the most common, accounting for about one-third of all cases of oxidative phosphorylation (OXPHOS) defects [1, 2]. CI, located in the inner mitochondrial membrane, is the largest of the five complexes. It is composed of 45 subunits, 7 encoded by mitochondrial DNA and 38 that are nuclear encoded [2]. CI functions to liberate and transfer electrons from NADH to ubiquinone, extracting energy to drive proton translocation across the inner mitochondrial membrane and thereby creating an electromechanical proton gradient that can be used in the last step of the respiratory chain for the synthesis of ATP, by ATP synthase [3]. Despite enormous progress in recent years in our ability to identify the genetic basis of inherited diseases, mainly due to the introduction of next-generation sequencing technologies into medical practice, cases of CI deficiency still remain undiagnosed [4]. In general, the clinical presentation of CI deficiency includes Leigh or Leigh-like syndrome, encephalomyopathy, cardiomyopathy, leukodystrophy, and hepatopathy [1]. Given the complexity of the respiratory chain’s multimeric complexes, and in particular CI, its assembly is a delicate, highly concerted process that involves the correct localization and function of all of the mitochondrial and nuclear encoded subunits. Any protein that has a role in the formation or stability of the respiratory chain’s macrocomplexes may be considered an assembly factor. More than a dozen CI assembly factors are already known, each encoded by a specific nuclear gene [1].

Biallelic pathogenic variants in the gene *NDUFAF2*, encoding the nuclear CI-assembly protein NDUFAF2, were initially reported to cause progressive encephalopathy beginning in infancy [5]. Since that initial report, only a few additional studies have described patients with loss-of-function mutations in *NDUFAF2* [6–10]. Collectively, those patients typically presented with infantile-onset nystagmus and progressive optic atrophy associated with growth retardation and global developmental delay, episodes of acute encephalopathy, and respiratory failure, resulting in early lethality.

In this study, we present four new cases from two separate families manifesting a progressive neuro-ophthalmological phenotype caused by a large homozygous intragenic deletion in *NDUFAF2* that results in the absence of NDUFAF2 protein. In addition, we summarize the clinical, radiological, biochemical, and genetic features of these patients and the previously described cases to further emphasize the distinctive phenotype associated with loss-of-function mutations in *NDUFAF2*.

## Patients and methods

### Patient enrollment and clinical data collection

Four female patients (age range 15 months to 5 years) residing in Northern Israel, with the same homozygous pathogenic variant in *NDUFAF2* were recruited to the current study. We collected their clinical, genetic, and neuroradiological data, including information related to neurodevelopment, growth parameters, ophthalmology, dysmorphology, and evolution of neuroimaging when available. The study was approved by the local (Emek Medical Center) ethics committee review board (EMC-118–22).

### Biochemical assay of OXPHOS

Enzymatic activities of the five OXPHOS complexes were determined in isolated skin fibroblast mitochondria from patient 1 (P1) by spectrophotometric methods, as previously described [11]. The values were expressed relative to the activity of cytochrome *c* oxidase and/or citrate synthase. Fibroblasts of the other three patients were not available for this analysis.

### Primary fibroblast cultures

Primary skin fibroblasts from P1 and age-matched controls were isolated and propagated under standard conditions in high-glucose Dulbecco’s Modified Eagle Medium supplemented with 10% (v/v) fetal bovine serum (FBS), 2 mM L-glutamine, 100 U/ml penicillin, and 100 mg/ml streptomycin (Biological Industries, Beit Haemek, Israel).

### Western blot assays

For analysis of protein-expression levels, cells were washed twice with FBS and lysed in urea sample buffer (10 M deionized urea, 1% sodium dodecyl sulfate, 10% glycerol, 60 mM Tris pH 6.8, and 5% β-mercaptoethanol). Total protein concentrations were equalized and samples were run on 10% SDS-PAGE gels. Gels were transferred to nitrocellulose membranes and probed with primary and secondary antibodies (goat anti-mouse/rabbit peroxidase, 1:5000, Rockland KPL, Gaithersburg, MD) against proteins of interest. Chemiluminescent imaging was performed using a G-Box imaging system (Syngene) and densitometry was performed using ImageJ (NIH). Densitometry quantification was normalized using alpha-tubulin as a loading control. Primary antibodies for the western blot were: rabbit anti-human NDUFAF2 antibody (diluted 1:1000, PA5-63,019, ThermoFisher Scientific); anti-VDAC1 antibody (diluted 1:1000, #ab15895, Abcam); and monoclonal mouse anti-human alpha-tubulin (diluted 1:4000, #T5168, Sigma-Aldrich).

### Chromosome microarray analysis (CMA)

CMA was performed on DNA samples of three of the probands and their parents (two couples), using Affymetrix CytoScan 750 K arrays according to the manufacturer’s instructions. The procedure included genomic DNA extraction, digestion, and ligation, PCR amplification, PCR product purification, quantification, and fragmentation, labeling, array hybridization, washing, and scanning. Data were analyzed with Chromosome Analysis Suite software version 4.1.0.90 (Affymetrix).

## Results

### Case reports

#### Patient 1 (P1)

The patient is the first female offspring born to healthy first-degree cousins of Arab Muslim descent. The pregnancy was remarkable for intrauterine growth retardation noted during the third trimester. She was born prematurely at 34 weeks of gestation with a birth weight of 1640 g (z-score − 2.32) and birth head circumference of 28.5 cm (z-score − 2.14). Her neonatal course at the neonatal intensive care unit (NICU) was marked by prolonged neonatal jaundice that was successfully treated with phototherapy. Initial cardiac echocardiogram after birth revealed mild concentric hypertrophy of the left ventricle along with mild pulmonary stenosis and moderate size atrial septum defect. Initial brain ultrasound was normal, as were routine investigations for congenital intrauterine infection. She was discharged at the age of 30 days with no further complications.

**Fig. 1:**
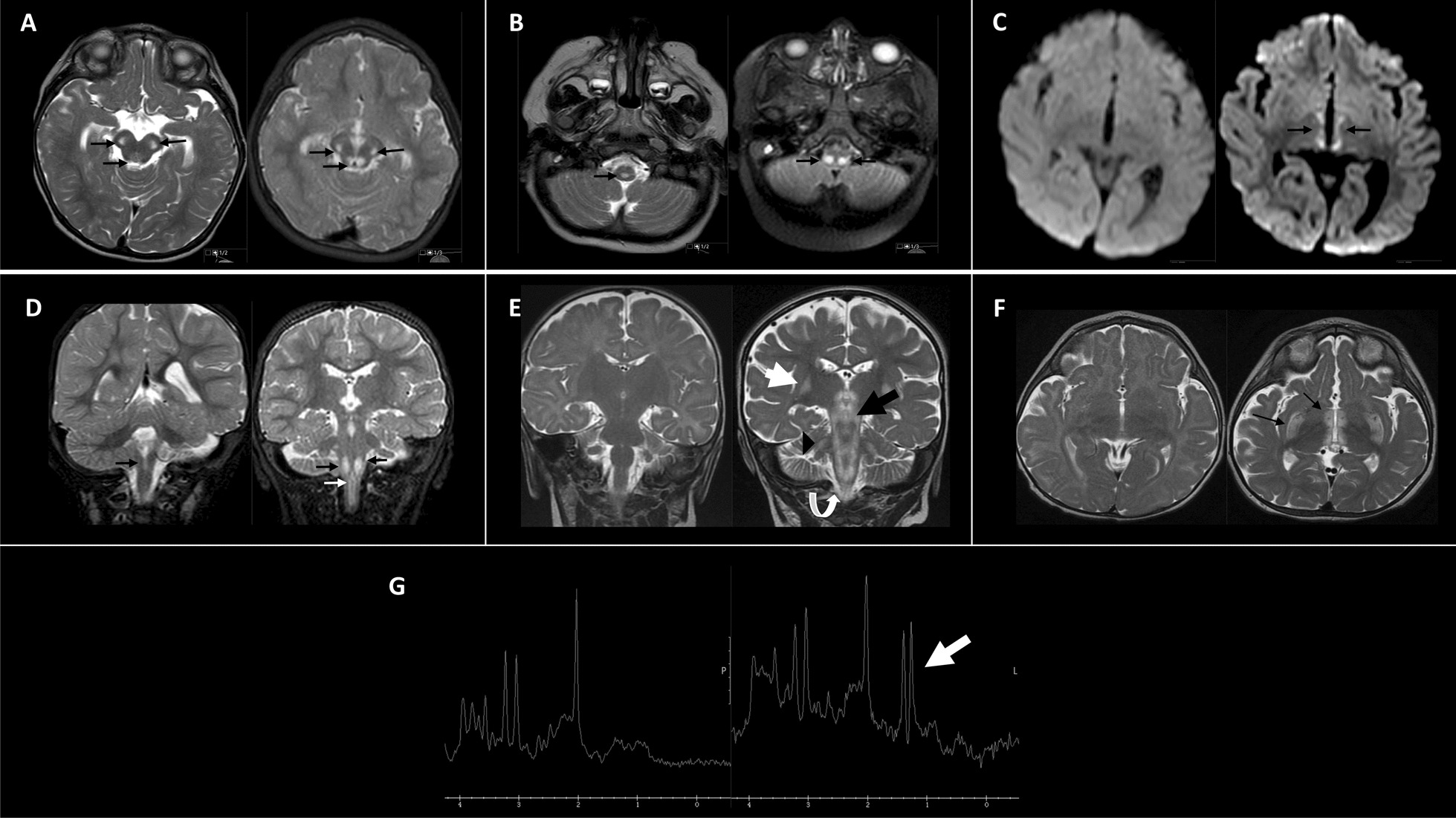
MR images of P1 and P4. P1 had two studies performed, at 16 months (**A**–**D**, left panels) and 30 months (**A**–**D**, right panels) of age. **A** Axial T2 images at the midbrain level with hyperintense lesions involving the cerebral peduncles and mild hyperintensity involving the periaqueductal gray matter (arrows). Follow-up (right panel) shows marked increase in the intensity surrounding the aqueduct (arrows). **B** Axial T2 images at the level of the medulla oblongata demonstrating hyperintensity involving the central medulla (arrow). The later scan (right panel) shows two additional hyperintense lesions involving the lateral portions of the medulla as well (arrows). **C** Diffusion-weighted images displaying restricted bilateral symmetrical diffusion of the medial thalamus in the later image (right panel, arrows) which did not exist in the earlier scan (left panel). **D** T2 coronal images exhibiting involvement of the central medulla (black arrow) whereas the follow-up image (right panel) shows additional increased intensity on the lateral portion of the medulla and a new long lesion involving the upper spinal cord (white arrow). P4 had sequential MR images, performed at 6 months (E–G, left panel) and 16 months (E–G, right panel) of age. **E** T2 coronal images. The later scan (right panel) shows bilateral symmetrical hyperintense lesions involving the putamen (white arrow), brainstem (black arrow), cerebellum (black arrowhead) and upper cervical cord (white curved arrow) **F** Axial T2 scans demonstrating bilateral symmetrical hyperintense lesions in the putamen and medial thalamus evident only on the later scan (black arrows in right panel). **G** MR spectroscopy centered at the thalamus exhibiting a lactate peak at 1.3 ppm which appeared only on the later assay (white arrow, right panel)

At the age of 6 months, she presented with intermittent bilateral horizontal nystagmus. Her disease course was remarkable for moderate symmetrical but stable growth retardation with microcephaly (weight and head circumference below the 3rd centile) and mild global developmental delay. At the age of 8 months, she was only able to rotate from back to belly; she was unable to sit, but she could grab objects and switch them from one hand to the other and put them in her mouth, and she babbled monosyllables. At the age of 16 months, she was able to sit steadily and to crawl, but could only walk two steps, with assistance. Initial brain magnetic resonance imaging (MRI) at the age of 16 months revealed abnormal T2-weighted symmetrical hyperintense signals at the cerebral peduncles and the medulla oblongata (Fig. 1A–D, left panels). At the age of 18 months, she was admitted for acute gastroenteritis. Her initial examination revealed severe growth retardation, moderate global developmental delay, microcephaly, truncal hypotonia, bilateral nystagmus with intermittent strabismus, and facial dysmorphic features dominated by bird-like facies with a broad nasal bridge. Repeated echocardiogram was normal, and in particular, there was no sign of cardiac hypertrophy. Ophthalmological examination at 18 months displayed bilateral temporal pallor compatible with evolving optic nerve atrophy. The patient started to walk independently and to utter her first words at the age of 2 years. Thereafter, she was admitted several times due to trivial febrile illnesses, expressing varying degrees of lethargy and recurrent episodes of apnea, as well as exacerbation of her nystagmus and limitation of both eyes’ movements. Serum biochemistry, lactate and ammonia, as well as arterial blood gases, were normal. In particular, urinary organic acid, serum amino acids, and acylcarnitine profiles were normal. Following these admissions, her disease progressed; further neurological deterioration manifested with truncal ataxia, the appearance of intention tremor, advanced motor disability, and rapid loss of previously acquired developmental milestones. In addition, she required the insertion of a gastrostomy tube for feeding. At the age of 30 months, the patient was admitted due to an episode of prolonged apnea at home which necessitated full resuscitation and mechanical ventilation. Repeated brain MRI revealed further progression with the appearance of new bilateral T2 hyperintense signals located in the brainstem and involving almost all of the medulla oblongata, and extending to the upper section of the cervical spinal cord and to the medial thalamus (Fig. 1A–D, right panels). The patient gradually developed severe hypertension that was partially controlled by aggressive medical treatment with clonidine (an alpha-2 receptor agonist) and angiotensin-converting enzyme inhibitors. In addition, she developed hyperphagia and significant weight gain within the next 2 months that were attributed to brainstem dysregulation. Repeated echocardiography showed hypertrophic cardiomyopathy that was stabilized with beta blockers. Her disease rapidly deteriorated with multiple episodes of central apneas that required tracheostomy implementation. The patient died at the age of 3.5 years.

### Patient 2 (P2)

This female patient is the twin sister of patient 3 (P3). Both are the first offspring of healthy first-degree cousins of Arab Muslim descent with no known family relation to P1. She was born at 34 weeks of gestation due to prolonged rupture of the membranes. Her birth weight was 1270 g (z-score − 2.92) and her birth head circumference was 26 cm (z-score − 3.10). Her course at the NICU was remarkable for mild respiratory distress syndrome that resolved with no respiratory sequelae and no ventilatory assistance. Initial brain ultrasound at the NICU was normal. She first presented at the age of 9 months with new-onset strabismus and intermittent nystagmus. At the age of 10 months, she was admitted to the pediatric department due to recurrent apneic episodes. On admission, her physical examination revealed normal vital signs, mild truncal hypotonia, symmetrical growth retardation, mild microcephaly, and facial dysmorphism with a triangular face and broad nasal bridge. Her cardiological examination, including echocardiography and ECG, was normal. Electroencephalographic (EEG) examination was unremarkable and in particular, did not show epileptic discharges or generalized slow waves consistent with encephalopathy. Follow-up neurological assessment at the age of 11 months revealed exacerbation of truncal hypotonia and new appearance of lower limb hypertonia. At the age of 18 months, she was admitted due to fever, respiratory distress, and lethargy. She was diagnosed with pneumonia and was treated with intravenous antibiotics but continued to deteriorate, with the development of hypoxia and metabolic acidosis. Her ophthalmological examination revealed bilateral strabismus with no evidence of optic atrophy or ophthalmoplegia. Due to multiple central apneic episodes, she required mechanical ventilation. Despite aggressive supportive treatment, she developed severe lactic acidosis and required continuous intravenous bicarbonate administration. Her course at the ICU continued to deteriorate with multiorgan failure and she succumbed at the age of 19 months.

### Patient 3 (P3)

This female baby is the twin sister of P2. Her birth weight was 1450 g (z score − 2.50) and her birth head circumference was 28 cm (z score − 2.38). Her neonatal course at the NICU was marked by prolonged jaundice necessitating phototherapy, and mild respiratory distress syndrome. Brain sonography was normal. She initially presented at the age of 5 months with new-onset strabismus and bilateral ophthalmoplegia dominated by limitation of her lateral gaze. In addition, she had progressive growth retardation, microcephaly, and facial dysmorphism, similar to her twin sister. She had no regular medical follow-up and she was documented to have mild global developmental delay but with no formal assessment. At the age of 15 months, she presented to the pediatric emergency room with acute pharyngitis and was discharged. Two weeks later she was found at home with no pulse and no breathing. Sudden infant death was presumed, but the parents refused an autopsy, and she had no genetic or metabolic analyses.

### Patient 4 (P4)

This female patient is the younger sister of P2 and P3. The parents refused prenatal testing for the known familial pathogenic variant. During pregnancy, intrauterine growth retardation, microcephaly, and thin corpus callosum were noted on a prenatal sonograph. Fetal echocardiogram was normal. Prenatal brain MRI at 30 weeks gestation confirmed a thin corpus callosum, without additional brain anomalies. She was born at 39 weeks by normal vaginal delivery. Her birth weight was 2485 g (z-score − 2.0) and her birth head circumference was 32.5 cm (z score − 1.5). Genetic assessment after birth confirmed homozygosity for the familial pathogenic variant. Her examination at the age of 3 months showed stable growth retardation and facial dysmorphism similar to both of her deceased sisters. In particular, neurological examination was normal, and her ophthalmological examination was unremarkable. Specifically, she had no strabismus or nystagmus. On follow-up examination at the age of 5 months, she had new-onset right eye strabismus and initial appearance of nystagmus. Brain MRI and MR spectroscopy (MRS) at the age of 6 months were normal. In particular, she had a normal lactate peak on MRS (Fig. 1E–G, left panels). At 7 months, disease progression had already occurred, with episodes of restlessness, disorganized hand movements with gentle tremor, and increased tone of her upper and lower limbs. Evolving global developmental delay was noted with inability to rotate from back to belly, lift her chest against gravity, and grab an object. At 11 months of age, she was able to sit for 30 s and crawl minimally. At the age of 1 year, she was admitted due to pneumococcal bacteremia. She had hypertension, her echocardiography revealed a hyperdynamic heart, and she started treatment with beta blockers. Thereafter, she had recurrent admissions due to apneic episodes and generalized neurological deterioration which were associated with febrile illnesses. Metabolic acidosis was present during these admissions with mild elevation of serum lactate. Repeated brain MRI at the age of 16 months showed abnormal extensive hyperintense signal involving the medulla oblongata, pontine tegmentum, upper cervical cord, putamina, dentate nuclei, medial thalami, and cerebellar peduncles (Fig. 1E, F, right panels). Brain MRS showed increased lactate peak at the level of the thalamus that was absent in the initial study (Fig. 1G). Her disease deteriorated and she required the insertion of a gastrostomy feeding tube. Due to prolonged episodes of apnea and chronic pCO_2_ accumulation, she also required a tracheostomy for assisted mechanical ventilation. She gradually developed chronic hypertension that was attributed to brainstem malfunction and which was only partially controlled by clonidine and diuretics. On her last follow-up, at the age of 4.5 years, the patient was in a vegetative state; she is bedridden with severe central hypotonia and has no communication with her environment. She is fed by gastrostomy tube and supported at home by mechanical ventilator. She has had multiple admissions to the pediatric ICU due to recurrent pneumonia and respiratory failure.

#### CMA

**Fig. 2:**
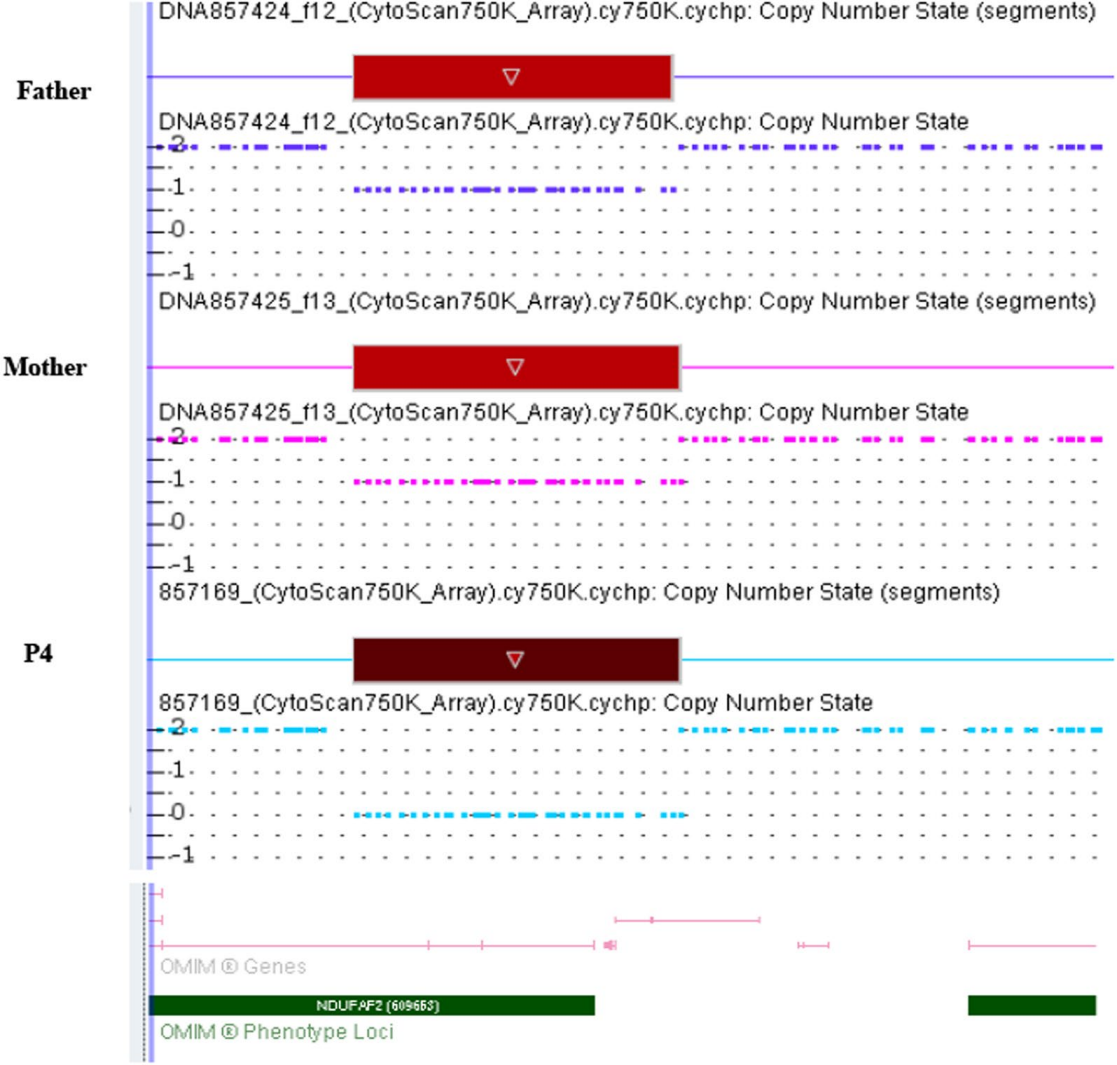
Chromosome microarray analysis (CMA) of P4 and her parents. Interstitial deletion of 142 kb is seen in a homozygous state in the patient (lower panel) and in a heterozygous state in both parents (upper and middle panels)

CMA results showed a homozygous 142-kb partial interstitial deletion of chromosome arr[GRCH37] 5q12.1 (60,325,346–60467500) × 0 in P1, P2, and P4, and a heterozygous 142-kb deletion of chromosome arr[GRCH37] 5q12.1 (60,325,346–60467500) × 1 in their parents. The deletion omits exons 2–4 of *NDUFAF2* (Fig. 2).

### Biochemical OXPHOS assay

Enzymatic activities of all five OXPHOS complexes of P1 revealed a significant decrease of CI in the patient’s fibroblasts (42% of the lowest control value), whereas all other complex activities were within the normal range. The activity of citrate synthase was above the highest reference value, suggesting a mitochondrial compensatory effect (Table 1).

**Table 1:** OXPHOS enzyme activities in cultured skin fibroblasts from P1

OXPHOS enzymatic activity	Fibroblast mitochondria		
	Patient	Control range	Units
Complex I	118	279–1076	mU/U COX
Complex II (SDH)	786	375–2692	mU/U COX
Complex II + III	607	325–649	mU/U COX
Complex III	1737	623–3534	mU/U COX
Complex IV (COX)	474	288–954	mU/U CS
Complex V (ATP synthase)	1244	480–2705	mU/U COX
Citrate synthase (CS)	602	151–449	mU/mg per min

### Western blot analysis of NDUFAF2

**Fig. 3:**
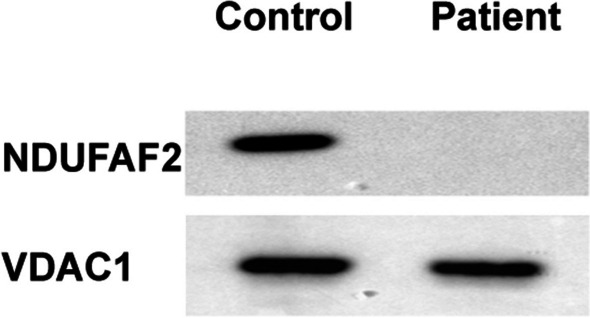
Immunoblot analysis with anti-NDUFAF2 antibody showing its complete loss in P1 compared to an age-matched control. Anti-VDAC1 antibody was used as a mitochondrial protein control

Immunoblots of P1’s fibroblasts using anti-NDUFAF2 antibodies revealed complete absence of this mitochondrial protein, in contrast to an age-matched control. Anti-VDAC1 antibody, used as a mitochondrial protein control, showed a normal signal (Fig. 3).

## Discussion

In the current study, we describe three patients (P1, P2, P4) from two separate families who presented with infantile onset nystagmus and evolving optic atrophy, and progressive neurodegeneration with profound brainstem disruption, due to a homozygous loss-of-function pathogenic variant in *NDUFAF2*. Another patient (P3), a sibling of two genetically confirmed patients (P2 and P4) with a rather similar phenotype, died abruptly before any genetic investigations could be performed. However, given the similar clinical course and family history, P3 may be regarded as suffering from the same disease and therefore was included in our patients’ cohort. All three patients who had genetic analyses were homozygous for the same chromosomal microdeletion that encompassed a large intragenic deletion in *NDUFAF2* containing three of the four *NDUFAF2* exons. As expected, western blot analysis of P1’s fibroblasts revealed that this deletion results in complete loss of NDUFAF2 protein. In agreement, given the critical role of NDUFAF2 in CI assembly [12], isolated CI activity was found to be significantly reduced in these fibroblasts. Taken together, we showed that our patients’ phenotype can be directly attributed to NDUFAF2 deficiency.

In general, all of our patients displayed moderate prenatal growth retardation. They initially presented with either nystagmus or strabismus, or both, within the first months of life, indicating cranial nerve impairment at the brainstem level. One patient achieved unassisted ambulation and initial speech abilities (P1) and the other three achieved unassisted sitting. However, global deterioration with loss of already-attained developmental milestones was the rule in all patients within the second or third year of life. Life-threatening apnea episodes occurred in all patients, which probably resulted in the sudden death of P3, whereas the other patients were successfully resuscitated. Two patients (P1, P4) had consecutive brain MRIs which revealed typical progressive lesions located primarily in the brainstem and later also involving the thalami, basal ganglia, cerebellum, and upper cervical spinal cord. Taken together, our patients fulfilled the clinical and neuroradiological diagnostic criteria of Leigh syndrome [13], a clinical entity with highly variable genetic heterogeneity involving pathogenic variants in two genomes (nuclear and mitochondrial) [14]. Defects in CI are the leading biochemical cause of Leigh syndrome [15] and NDUFAF2, an established CI-assembly factor, is among the various genetic causes resulting in this syndrome [1, 14].

To date, only 14 patients with loss-of-function mutations in *NDUFAF2* (including our 4 patients) have been reported. In Table 2, we summarize their clinical, biochemical, neuroradiological, and genetic characteristics [5–10, 16]. Notably, in three of the historical patients, only the genetic data were included without further clinical details, except for the clinical definition of Leigh syndrome [16]. Overall, most of the patients presented with ophthalmological impairments (mainly nystagmus) within the first months of life with later onset of global developmental delay, ataxia, optic atrophy, and gradual loss of developmental milestones. Most patients experienced further neurological deterioration, becoming bedridden and requiring gastrostomy feedings. Apnea episodes were common, leading to further respiratory deterioration and ventilator dependency for most of those who survived these episodes. Of note, seven patients were able to sit independently and four of them acquired independent walking, with three achieving initial speech abilities. Most of the patients for whom a detailed clinical description is available [5–10] died or were on a ventilator within the first 3 years of life, except for one patient who died at the age of 13 years [5], indicating an overall grave prognosis.

**Table 2:** Clinical, biochemical, neuroradiological, and genetic features of patients with loss-of-function mutations in NDUFAF2

Patient #	1	2	3	4	5	6	7	8	9	10	11	12	13	14
Reference	Ogilivie et al. [5]	Barguthi et al. [6]	Barguthi et al. [6]	Hoefs et al. [8]	Janssen et al. [9]	Herzer et al. [7]	Calvo et al. ([6]	Calvo et al. [16]	Calvo et al. [16]	Ghaloul Gonzalez et al. [10]	Current study (P1)	Current study (P2)	Current study (P3)	Current study (P4)
Gender	F	F	M	M	F	M	NA	Na	Na	M	F	F	F	F
IUGR	NA	NA	NA	NA	Yes	NA	NA	Na	Na	NA	Yes	Yes	Yes	Yes
Consanguinity	No	Yes	Yes	Yes	Yes	No	NA	Na	NA	NA	Yes	Yes	Yes	Yes
Age at presentation (first symptom)	12 m (N/ & ataxia)	20 m (apnea)	8 m (HY, DD, N, OA)	5d (HCM)	Birth (severe GR)	14 mo (N/DD)	NA	NA	NA	Infancy (N, DD, HY, DD)	6 mo (N)	9 mo (N,S)	5 mo (S,GR)	5 mo (S,N)
Age at death	13y	2y	21 mo	11 mo	14 m	27 mo	NA	NA	NA	19 mo	3.5y	19 mo	15mo	Alive at 5y
Optic atrophy (age at onset)	3 y	NA	8 mo	6 mo	NA	26 mo	NA	NA	NA	NA	18 mo	No	NA	3y
Nystagmus (age at onset)	12 mo	20 mo	8 mo	NA	NA	14 mo	NA	NA	NA	Yes	6 mo	9 mo	NA	5 mo
Strabismus (age at onset)	NA	20 mo	NA	NA	NA	NA	NA	NA	NA	NA	18 mo	9 mo	5 mo	5 mo
GR (age at onset)	3y	No	NA	NA	Birth	NA	NA	NA	Na	Yes	birth	10 mo	5 mo	3 mo
DD (age at onset)	3y	No	8 mo	Birth	Birth	NA	NA	NA	NA	Yes	6 mo	10 mo	9 mo	7 mo
Apnea (age at onset)	8y	20 m	18 mo	8 mo	NA	26 m	NA	NA	NA	15 mo	18 mo	10 mo	15 mo	12 mo
Gastrostomy (age)	8y	No	18 m	NA	NA	NA	NA	NA	NA	No	2y	No	No	20 mo
Mechanical ventilation (age)	8y	No	18 m	8 mo	NA	26 mo	NA	NA	NA	15 mo	3y	18 m	No	26 mo
Beginning to sit (age)	Age appropriate	Yes	No	No	No	yes	NA	NA	NA	NA	16 mo	12 mo	12 mo	11 m
Beginning to walk (age)	Age appropriate	Yes	No	No	No	22 m	NA	NA	NA	No	2y	No	No	No
Initial words (age)	Age appropriate	Yes	No	No	No	NA	NA	NA	NA	NA	18 m	No	No	No
Ser Lactate	N	Normal	Normal	NA	↑	↑	NA	NA	NA	↑	Normal	↑	ND	↑
Csf Lactate	↑	Normal	Normal	NA	NA	↑	NA	NA	NA	NA	NA	↑	ND	Na
Complex I activity in tissue % of normal (tissue)	20% (muscle)	36% (fibroblast	24% (fibroblast	36% (Fibroblast) 20% (muscle)	45% (fibroblast	12% (muscle) 59% (fibroblast	NA	NA	NA	NA	42% (fibroblast	ND	ND	ND
NDUFAF2 mutation	c.182C > T (hom) p.R45X	c.1A > T (hom) p.M1L	c.1A > T (hom) p.M1L	c.114C > G (hom) p.Y38X	Triple gene deletion including NDUFAF2 (hom)	c.9G > A (p.W3X) (hom)	C.221G > A (p.W74*) (hom)	C.103delA (p.I35Sfsx17) (hom)	C.103delA (p.I35Sfsx17) (hom)	NDUFAF2 gene deletion (hom)	NDUFAF2 intragenic deletion (hom)	NDUFAF2 intragenic deletion (hom)	ND	NDUFAF2 intragenic deletion (hom)
Brain MRI (age)	Normal (**12mo**) abnormal signal: BS,T, CS (**3.5y**) Generalized atrophy (**10y**)	Abnormal signal BS, CE (**20 mo**)	Abnormal signal BS, CE (**19 mo**)	Cer atrophy, CC hypoplasia (**4 mo**)	NA	Abnormal signal BS, CS (**27 mo**)	NA	NA	NA	Abnormal signal BS, CE, CS, BG (**15mo**)	Abnormal signal BS (18 mo) Abnormal signal BS, T, CS (**18 mo**)	ND	ND	Normal (**6 mo**) Abnormal signal BS, T,CS,CE, BG (**16 mo**)

*NA* Not available, *ND* Not done, *BS* Brainstem, *T* thalamus, *CS* cervical spine, *BG* Basal ganglia, *CE* Cerebellum, *Cer-cerebral* CC-corpus callosum, *d* Day, *y* Years, *mo* Months, hom-homozygous, *HY* Hypotonia, *N* Nystagmus, *S* Strabismus, *OA* Optic atrophy, *GR* Growth retardation, *IUGR* Intra-uterine growth retardation, *HCM* Hypertrophic cardiomyopathy, *ser* serum, *csf* Cerebrospinal fluid

Neuroradiology signifies a major hallmark in NDUFAF2-deficient patients. Barghuti et al. [6] suggested a unique characteristic distribution of MRI abnormalities involving mainly the brainstem, cerebellum, and upper cervical spinal cord with sparing of the basal ganglia. However, later reports, including the current study, showed that the neuroradiology phenotype has a typical progressive course, in keeping with disease development. In P4, initial cranial MRI at 6 months was unremarkable despite the occurrence of initial clinical signs of the disease, suggesting relative neuroradiological delay. In P1 initial cranial MRI at the age of 16 months showed relatively minor abnormalities (hyperintense T2 signal) in the midbrain and the central medulla. In both patients later imaging showed further advance involving the entire brainstem (P1 & P4), the cerebellum (P4), the basal ganglia (mainly the putamen) (P4), medial and lateral thalamus (P1 & P4) and the upper cervical spinal cord (P1 & P4) (Fig. 1). In summary, NDUFAF2-associated Leigh syndrome has a distinctive neuroradiological course and progression that differ somewhat from the classical Leigh syndrome pattern with radiological lag compared with the clinical symptoms and inter individual variability even in patients with the same genotype. In the current study, we show for the first time an abnormally elevated lactate peak in MRS (P4). Of note, the initial scan in this patient at 6 months of age showed a normal lactate peak, suggesting that this abnormality is also dependent on disease progression.

Biochemical studies are largely noninformative in NDUFAF2-associated Leigh syndrome. Serum and cerebrospinal fluid lactate levels may be elevated but were within normal levels in several cases (Table 2). Furthermore, in the current study, serum lactate fluctuated inter individually between the normal range and mild–moderate elevations, in agreement with its being a nonspecific and unreliable diagnostic biomarker. Nevertheless, elevated lactate levels in the cerebrospinal fluid, particularly when associated with an abnormal MRI, raise high suspicion of a mitochondrial disorder and indicate further targeted genetic analysis.

Genetic analyses of *NDUFAF2* in all patients reported till now have revealed homozygous pathogenic variants that lead to complete loss of function. Of the 9 pathogenic variants identified to date in the 14 reported patients (Table 2), 4 were nonsense resulting in protein truncation, 3 resulted in complete or near complete *NDUFAF2* deletion, 1 frameshift mutation was predicted to prematurely truncate the protein, and 1 mutation abolished the initiating Met, resulting in untranslated protein [6]. When analyzed by western blot, NDUFAF2 was absent in all cases tested, confirming complete loss of protein as the causative disease [5, 6, 8, 9 and the current report]. Whether patients with partial NDUFAF2 activity due to missense mutations, for example, present a milder phenotype has yet to be determined. When tested, CI activity was found to be reduced in all patients (fibroblasts or skeletal muscle) [5–9 and the current report]. However, in clinical practice, OXPHOS activity is no longer required for a diagnosis of NDUFAF2-associated Leigh syndrome, and clinicians can rely on the typical clinical and neuroradiological phenotype associated with confirmatory *NDUFAF2* loss-of-function variants. Biochemical OXPHOS analysis may be reserved for cases with clinical and/or genetic ambiguity.

## Conclusions

Pathogenic variants in *NDUFAF2* result in a distinctive clinical and neuroradiological form of Leigh syndrome characterized by progressive brainstem disruption with early lethality.

## Data Availability

All data generated or analysed during this study are included in this published article [and its supplementary information files].

## Abbreviations

OXPHOS: Oxidative phosphorylation
NADH: Ubiquinone oxidoreductase (complex I, CI)
CMA: Chromosome microarray analysis
NICU: Neonatal intensive care unit
MRI: Magnetic resonance imaging
MRS: MR spectroscopy
